# HCRP-1 regulates EGFR–AKT–BIM-mediated anoikis resistance and serves as a prognostic marker in human colon cancer

**DOI:** 10.1038/s41419-018-1217-2

**Published:** 2018-12-05

**Authors:** Feifei Chen, Lei Zhang, Jianqiang Wu, Fuchun Huo, Xin Ren, Junnian Zheng, Dongsheng Pei

**Affiliations:** 1 Jiangsu Cancer Biotherapy Institute, Xuzhou Medical University, Xuzhou, Jiangsu P. R. China; 2 Medical Imaging Department, Huaian‘ Second People‘s Hospital and The Affiliated Huaian‘ Hospital of Xuzhou Medical University, Huaian, Jiangsu P. R. China; 3 Jiangsu Center for the Collaboration and Innovation of Cancer Biotherapy, Cancer Institute, Xuzhou Medical University, Xuzhou, Jiangsu P. R. China; 4 Cancer Center, The Affiliated Hospital of Xuzhou Medical University, Xuzhou, Jiangsu P. R. China; 5 Pathological Department, Xuzhou Medical University, Xuzhou, Jiangsu P. R. China

## Abstract

Hepatocellular carcinoma-related protein-1 (HCRP-1), a subunit of mammalian endosomal sorting complex required for transport-I (ESCRT-I), is frequently downregulated in various kinds of malignant tumors. The role of HCRP-1 in colorectal cancer (CRC) remains unknown. We investigate the clinical value of HCRP-1 and its impact on anoikis in CRC. The negative expression of HCRP-1 was significantly correlated with tumor size (*P* *=* 0.033), PT status (*P* *=* 0.001), TNM stage (*P* *=* 0.039), and histological grade (*P* *=* 0.01). Univariate and multivariate analyses revealed that HCRP-1 was an independent prognostic factor for CRC (hazard ratio (HR) = 0.237, *P* < 0.001 for 5-year overall survival). In the in vitro assay, we found that HCRP-1 depletion resulted in cell anoikis resistance. Knockdown of HCRP-1 suppressed Bcl-2 interacting mediator of cell death (BIM) expression, with phosphorylation of AKT and p-FoxO3a, which was reversed by AKT siRNA or AKT inhibitor. Further analysis showed that loss of HCRP-1 obviously increased the activation of EGFR. Inhibition of EGFR blocked si-HCRP1-mediated phosphorylation of EGFR, AKT, FoxO3a, and BIM expression. Moreover, the in vivo results revealed that loss of HCRP-1 promoted cancer metastasis. Our findings implied that reduced HCRP-1 expression in CRC resulted in anoikis resistance and contributed to CRC metastasis and poor prognosis. These data may help design effective therapy targeting HCRP-1 pathway to control colon cancer growth and metastasis.

## Background

CRC is the second most common cancer in females and the third in males, and the fourth highest reason of cancer-associated deaths all over the world^1^. Although surgical resection is one of the most effective treatments for patients with CRC, 30% of patients still form tumor metastasis^2,3^. Hence, novel prediction, diagnosis, individualized medication, and evaluation of prognosis biomarkers are urgently needed for CRC.

EGFR belongs to the ErbB family of transmembrane receptor tyrosine kinases, which is widely distributed on the surface of mammalian cell membranes. It plays an important role in governing multiple cellular processes, containing cell proliferation, survival, and migration^4,5^. Studies show that EGFR alteration is involved in the pathogenesis and progression of many malignancies. In particular, hyperactivation of EGFR-dependent signal transduction usually accompanies tumor development and leads to unfavorable clinical prognosis^6^. Therefore, targeting the EGFR pathway constitutes a potential treatment modality for human cancers.

EGFR downregulation by the endosomal sorting process depends on receptor internalization via endocytosis, and subsequent vesicular shuttling toward one of three distinct cytoplasmic compartments^7^. Endosomal sorting complexes required for transport-I (ESCRT-I) is essential for sorting of ubiquitinated transmembrane proteins, such as EGFR, into internal vesicles of MVBs and subsequent degradation. HCRP-1, also called human vacuolar protein sorting 37 homolog A (hVps37A), is a subunit of mammalian ESCRT-1 and mediates the internalization and degradation of ubiquitinated membrane receptor EGFR^8^. The HCRP-1 gene is located on the short arm of chromosome 8, where loss of heterozygosity is often reported in many types of cancers, indicating its potential tumor suppressor role in cancers. Previous studies showed that the expression of HCRP-1 is decreased in hepatocellular carcinoma, breast cancer, oral cancer, and gastric cancer^9–13^. Decreased HCRP-1 expression is associated with activation of EGFR, and its expression has a significant impact on the prognostic value of EGFR expression in ovarian cancer. Moreover, silencing HCRP-1 induces an invasive phenotype in vitro and tumor growth in vivo^11^. Previously, we demonstrated that HCRP-1 is an independent prognostic factor for renal cell carcinoma and its absence promotes cell migration and invasion^14^. However, whether and how HCRP-1 functions in human CRC remain unknown.

In the current study, we evaluated the expression of HCRP-1 protein using TMA of CRC. The relationship between HCRP-1 expression and clinicopathological features was also examined. In addition, we showed a novel role for HCRP-1 in suppressing anoikis in human CRC cells. In vitro and in vivo studies confirmed that depletion of HCRP-1 promoted CRC metastasis and decreased BIM expression. Finally, we provided evidence that HCRP-1 knockdown negatively regulated BIM expression through the EGFR–AKT signaling pathway.

## Methods

### Patients and samples

A TMA for CRC was obtained from Shanghai Xinchao Biotechnology (China). It contained 80 matched normal tissues (NT) and 100 colon cancer tissues (CT). The follow-up time was ~7 years from the date of surgery to the date of death, or last visit. The detailed clinicopathologic features of patients are provided in Table 1. The tumor clinical stages and classification grade were obtained on the basis of the seventh American Joint Committee on Cancer (AJCC) classification.

**Table 1:** Association of HCRP-1 expression with clinical parameters in colon cancer patients

Variable	HCRP-1 staining			
	Negative (%)	Positive (%)	Total	*P*-value
All cases	42 (42.0)	58 (58.0)	100	
*Age (years)*				0.698
≤67	20 (43.5)	26 (56.5)	46	
>67	21 (39.6)	32 (60.3)	53	
*Gender*				0.211
Male	21 (36.2)	37 (63.8)	58	
Female	20 (48.8)	21 (51.2)	41	
*Tumor size (cm)*				0.033^*^
≤5	18 (32.1)	38 (67.9)	56	
>5	23 (53.5)	20 (46.5)	43	
*pT status*				0.001^*^
pT2	0 (0.0)	4 (100.0)	4	
pT3	21 (32.8)	43 (67.2)	64	
pT4	21 (65.6)	11 (34.4)	32	
*pN status*				0.072
pN0	17 (32.7)	35 (67.3)	52	
pN1	17 (47.2)	19 (52.8)	36	
pN2	8 (66.7)	4 (33.3)	12	
*pM status*				0.158
pM0	38 (40.0)	57 (60.0)	95	
pM1	4 (80.0)	1 (20.0)	5	
*TNM stage*				0.039^*^
I	0 (0.0)	4 (100.0)	4	
II	16 (34.0)	31 (66.0)	47	
III	22 (50.0)	22 (50.0)	44	
IV	4 (80.0)	1 (20.0)	5	
*Grade*				0.01^*^
G1	2 (14.3)	12 (85.7)	14	
G2	33 (42.9)	44 (57.1)	77	
G3	7 (77.8)	2 (22.2)	9	

*P-value < 0.05 shows statistical significance.

### Antibodies and reagents

Anti-HCRP-1 rabbit polyclonal antibodies were obtained from Proteintech (Wuhan, China). Rabbit monoclonal antibodies to AKT (#9272), p-AKT (Ser473, #9271), p-EGFR (Tyr1173, #2234), FoxO3a (#12829), p-FoxO3a (Ser253, #9466), Bcl-2 (#2872), and Mcl-1 (#4572) were purchased from Cell Signaling Technology (Shanghai, China). Antibodies against EGFR (sc-03-G), ERK (sc-271269), and p-ERK (Tyr204, sc-7382) were purchased from Santa Cruz (CA, USA). Mouse and rabbit anti-β-actin were obtained from Boster Biotechnology (Wuhan, China). Anti-BIM rabbit polyclonal antibody (BS64039) was ordered from Bioworld (Shanghai, China). AKT inhibitor, MK-2206 was obtained from Selleck (HOU, USA) and EGFR inhibitor, AG1478 was purchased from Abcam (Shanghai, China).

### Immunohistochemistry

Immunohistochemistry staining was performed as described previously^15^. The TMA slide was incubated with rabbit HCRP-1 antibody (1:50), anti-p-AKT (1:50), and p-EGFR (1:50) overnight at 4 °C, and diaminobenzidine (DAB; Zhongshan Biotech, Beijing, China) was used to produce a brown precipitate. For the immunohistochemistry staining evaluation, the immunoreactivity was assessed blindly by two independent observers using light microscopy (Olympus BX-51 light microscope), and the image was collected by Camedia Master C-3040 digital camera. The expression of HCRP-1 was graded as positive when 10% of tumor cells showed immunopositivity. Biopsies with 10% tumor cells showing immunostaining were considered negative^14,16^.

### Cell culture

Human CRC cell lines HCT116 and SW620 were obtained from the Shanghai Institute of Biochemistry and Cell Biology, Chinese Academy of Sciences (Shanghai, China). DMEM with 10% fetal bovine serum (Gibco, MEL, USA) was used as culture medium in HCT116 cells. SW620 cells were maintained in L-15 medium supplemented with 10% FBS. Cells were in a 37 °C humidified incubator with 95% air, 5% CO_2_.

### Transfection and SW620 stable cell line production

siRNAs for HCRP-1, AKT, and EGFR were obtained from Integrated Biotech Solutions (Shanghai, China). The sequences of siRNAs are as follows: HCRP-1, 5′-GACACUGUUUCUUCUUCAACA-3′ AKT, 5′-GAAGGAAGUCAUCGUGGCCTT-3′ EGFR, and 5′-CACAGUGGAGCGAAUUCCUTT-3′. Transfection with siRNA was performed using siLentFect Lipid Reagent (Bio-Rad, Hercules, CA, USA) according to the manufacturer’s instructions. The pcDNA3.1-control and pcPNA3.1-BIM expression plasmids were obtained from Integrated Biotech Solutions (Shanghai, China). Transfection of the pcPNA3.1-control and pcPNA3.1-BIM plasmids into the colon cancer cells was carried out using Lipofectamine 2000 transfection reagent (Invitrogen, Shanghai, China) following the manufacturer’s protocol.

SW620 cell lines were infected with lentivirus-packing HCRP-1 shRNA vector and control vector, respectively (IBS, Shanghai, China). Target cells were established by infecting with lentivirus about 2 days and then screened out with puromycin (Sigma, Shanghai, China) for 2 weeks.

### Anoikis and apoptosis assays

Transfection was performed as described previously. The cells were harvested 48 h after transfection with siRNA for HCRP-1. The rate of apoptosis cells was detected by staining with Annexin V-phycoerythrin (PE)/7-aminoactinomycin D (7-AAD) (BD, CA, USA) according to the manufacturer’s instructions. Briefly, cells were suspended in 200 μl of binding buffer. The cells were incubated with 10 μl of PE-Annexin V solution and 10 μl of 7-AAD at room temperature for 30 min, respectively. Then, cells were analyzed by FACScan flow cytometry (BD, CA, USA). For anoikis assay, transfected cells were seeded into six-well plates (Corning, NY, USA) with low-attachment surface (to avoid aggregating of cells) in normal growth media. Cells that did not adhere were submitted to flow cytometry detection.

### Western blot analysis

For CRC cells, cells transfected with siRNA were harvested from the plates in 48 h. Protein samples from cells were extracted in cell lysis buffer and then quantified using the BCA protein assay (KeyGEN Biotech, Nanjing, China). Samples were resolved by SDS-PAGE, then transferred to NC membranes (PALL, NY, USA), and incubated overnight at 4 °C with the primary antibody described earlier in Antibodies and Reagents. After incubation with peroxidase-coupled anti-mouse or anti-rabbit IgG antibody (1:1000, Zhongshan Biotech, Beijing, China) at 37 °C for 2 h, the specific proteins on NC membrane were detected on the Odyssey Two-Color Infrared Imaging System (LI-COR Biotechnology, Lincoln, Nebraska, USA). Each blot was analyzed in biological triplicate.

### Tumor metastases model in nude mice

Female BALB/c (6 weeks old) nude mice were obtained from Beijing Fukang Biotechnology (China). Animal experiments were approved by the Animal Care Committee of Xuzhou Medical University. To produce experimental metastasis, the mice were divided into two groups (*n* = 10, respectively) at random. After that, shCtrl-SW620 and shHCRP-1-SW620 cells (5.0 × 10^6^) were suspended in 200 μl of PBS and then injected into the tail vein of nude mice. Two months later, the two groups of mice were killed, their lungs were excised, and fixed with formalin in order to count metastatic nodules and analyze immunohistochemistry staining at the next step.

### Statistical analysis

The SPSS 17.0 statistical software (SPSS Inc., Chicago, IL, USA) was used for statistical analyses. Statistical calculations of the apoptosis, anoikis, and western blot data were performed using the Dunnett’s t test when two treatment groups were compared. For the colon cancer TMA, the χ^2^ test was used to evaluate the relations between HCRP-1 expression and clinicopathological features. Different expression of HCRP-1 between the carcinoma tissues and normal human colon tissues was analyzed by χ^2^ test, too. Survival curves were calculated using the Kaplan–Meier method, and differences between HCRP-1 expression and patient survival were evaluated by the log-rank test. Univariate and multivariate Cox regression analyses were used to analyze the independent effect of HCRP-1 and clinicopathologic parameters on survival. Only these variables owing more important significance (statistical standard: *P* < 0.05) in univariate analysis were selected to subsequent multivariate analysis. The results are described as the mean ± SD of at least three independent experiments. For all tests, a two-sided *P*-value < 0.05 was considered statistically significant.

## Results

### HCRP-1 expression and clinicopathological features in colon cancer

**Fig. 1:**
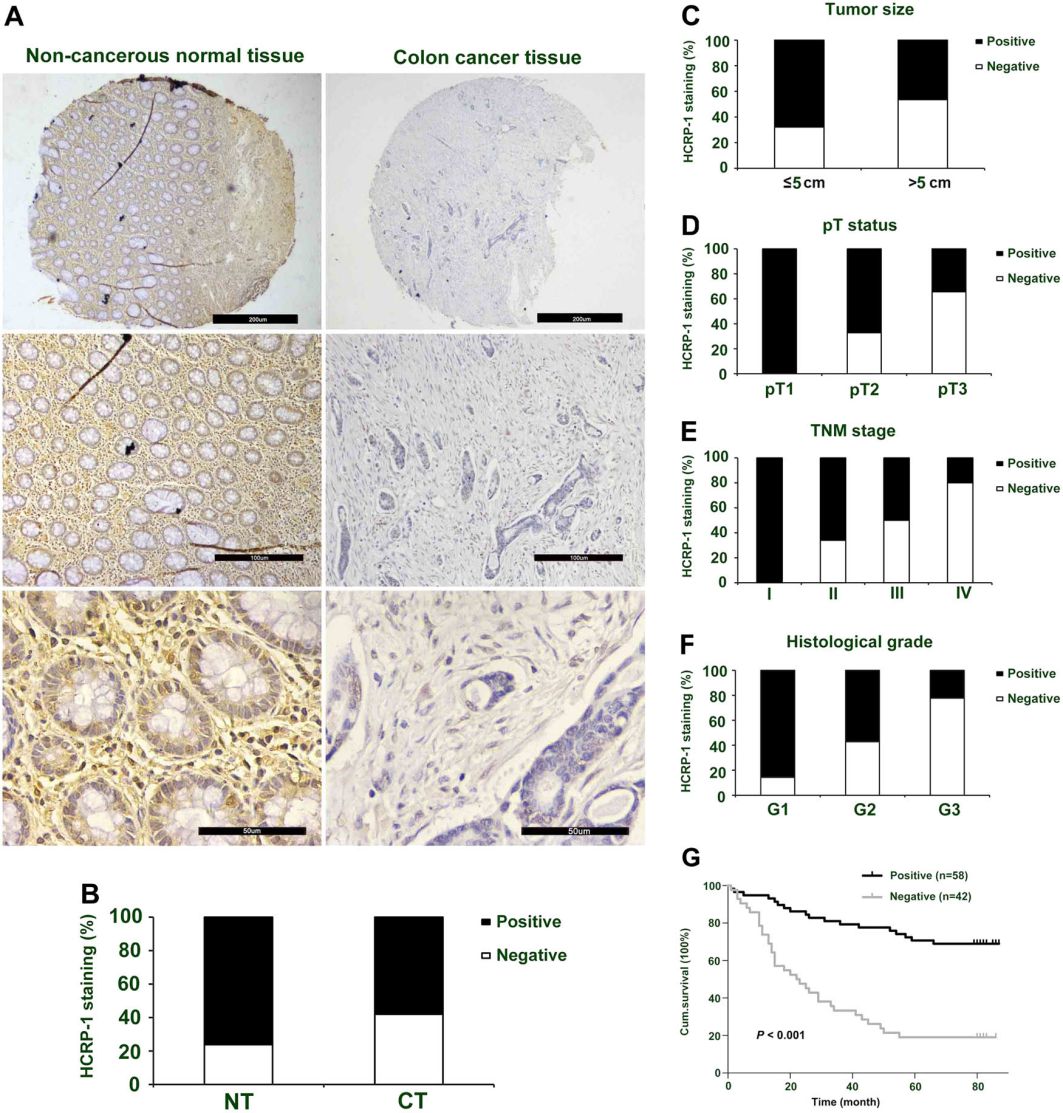
Association between HCRP-1 expression and clinicopathological characteristics in TMA of CRC. **a** Representative immunohistochemical photographs of HCRP-1 expression in CRC tissues, compared with NT (original magnification: top panel × 40, middle panel × 100, and bottom panel × 400). **b** HCRP-1 expression in CRC tissues was lower than that in para-cancer tissues (*P* *=* 0.01, χ^2^ test). **c** Negative expression of HCRP-1 had a significant correlation with tumor size (*P* *<* 0.05, χ^2^ test). **d** Negative expression of HCRP-1 had a significant correlation with pT status (*P* *<* 0.05, χ^2^ test). **e** Negative expression of HCRP-1 had a significant correlation with TNM stage (*P* *<* 0.05, χ^2^ test). **f** Negative expression of HCRP-1 had a significant correlation with histological grade (*P* *<* 0.05, χ^2^ test). **g** Kaplan–Meier curves of overall survival of patients according to HCRP-1 expression in 100 CRC patients (*P* *<* 0.001, log-rank test)

The expression of HCRP-1 protein was analyzed in TMA slide containing 100 colon cancer specimens and 80 noncancerous tissues by immunohistochemistry staining. The obvious cytoplasmic HCRP1 staining is shown in Fig. 1a. Of 100 CRC patients, positive HCRP-1 staining was detected in 58.0% (58 of 100 cases). However, in adjacent normal colon tissues, positive expression of HCRP-1 was recorded in 76.3% (61 of 80 cases) (Fig. 1b). Compared with noncancerous tissues, HCRP1 expression in CRC tissue was modest to weak (*P* *=* 0.01, χ^2^ test). These data suggest that HCRP-1 expression was low in colon cancer tissues.

The parameters of the study cohort and their correlation with HCRP-1 expression are shown in Table 1. Our data indicated that low expression of HCRP-1 was closely associated with tumor size (*P* = 0.033, Fig. 1c), pT status (*P* *=* 0.001, Fig. 1d), TNM stage (*P* *=* 0.039, Fig. 1e), and histological grade (*P* *=* 0.01, Fig. 1f). No significant differences were detected between HCRP-1 expression and other clinicopathologic characteristics, including age, gender, pN status, and pM status (*P* *>* 0.05).

### HCRP-1 and survival

To illuminate the prognostic impact of HCRP-1 in CRC, Kaplan–Meier analysis and log-rank test were employed to explore the relationship between HCRP-1 status and clinicopathologic parameters. The overall mortality events were 100. The median follow-up was ~7 years. Compared with those with increased expression of HCRP-1, patients with decreased HCRP-1 showed a lower overall survival rate by Kaplan–Meier survival analysis (*P* < 0.001, Fig. 1g).

In addition, univariate and multivariate analyses using the Cox proportional hazards showed that HCRP-1 could be useful as an independent prognostic marker in the 100 cases of colon cancer. Univariate analysis showed that HCRP-1 and pM status were significantly correlated with overall survival in colon cancer (HCRP-1: hazard ratio, 0.219, *P* < 0.001; pM status: hazard ratio, 5.142, *P* = 0.001). However, the multivariate analysis showed that HCRP-1 expression served as an independent prognostic factor (HCRP-1: hazard ratio, 0.237, *P* < 0.001) (Table 2).

**Table 2:** Univariate and multivariate Cox regression analyses in colon cancer patients

Variable	HCRP-1 staining			
	Univariate		Multivariate	
	HR (95% CI)	*P*-value	HR (95% CI)	*P*-value
*HCRP-1*		<0.001^*^		<0.001^*^
Negative	1.000 (reference)		1.000 (reference)	
Positive	0.219 (0.122–0.392)		0.237 (0.130–0.431)	
*Age (years)*		0.098		
≤67	1.000 (reference)			
>67	1.617 (0.916–2.854)			
*Gender*		0.351		
Male	1.000 (reference)			
Female	0.763 (0.432–1.347)			
*Tumor size (cm)*		0.709		
≤5	1.000 (reference)			
>5	1.110 (0.642–1.918)			
*pT status*		0.538		
pT2 + pT3	1.000 (reference)			
pT4	1.197 (0.676–2.119)			
*pN status*		0.082		
pN0	1.000 (reference)			
pN1 + pN2	1.627 (0.941–2.813)			
*pM status*		0.001^*^		0.054
pM0	1.000 (reference)		1.000 (reference)	
pM1	5.142 (1.984–13.326)		2.592 (0.985–6.820)	
*TNM stage*		0.052		
I + II	1.000 (reference)			
III + IV	1.725 (0.994–2.992)			
*Grade*		0.446		
G1	1.000 (reference)			
G2 + G3	1.392 (0.594–3.261)			

*HR* hazard ratio, *CI* confidence interval

*P-value  <0.05 shows statistical significance

### Effects of HCRP-1 downexpression on anoikis and apoptosis in CRC cell lines

**Fig. 2:**
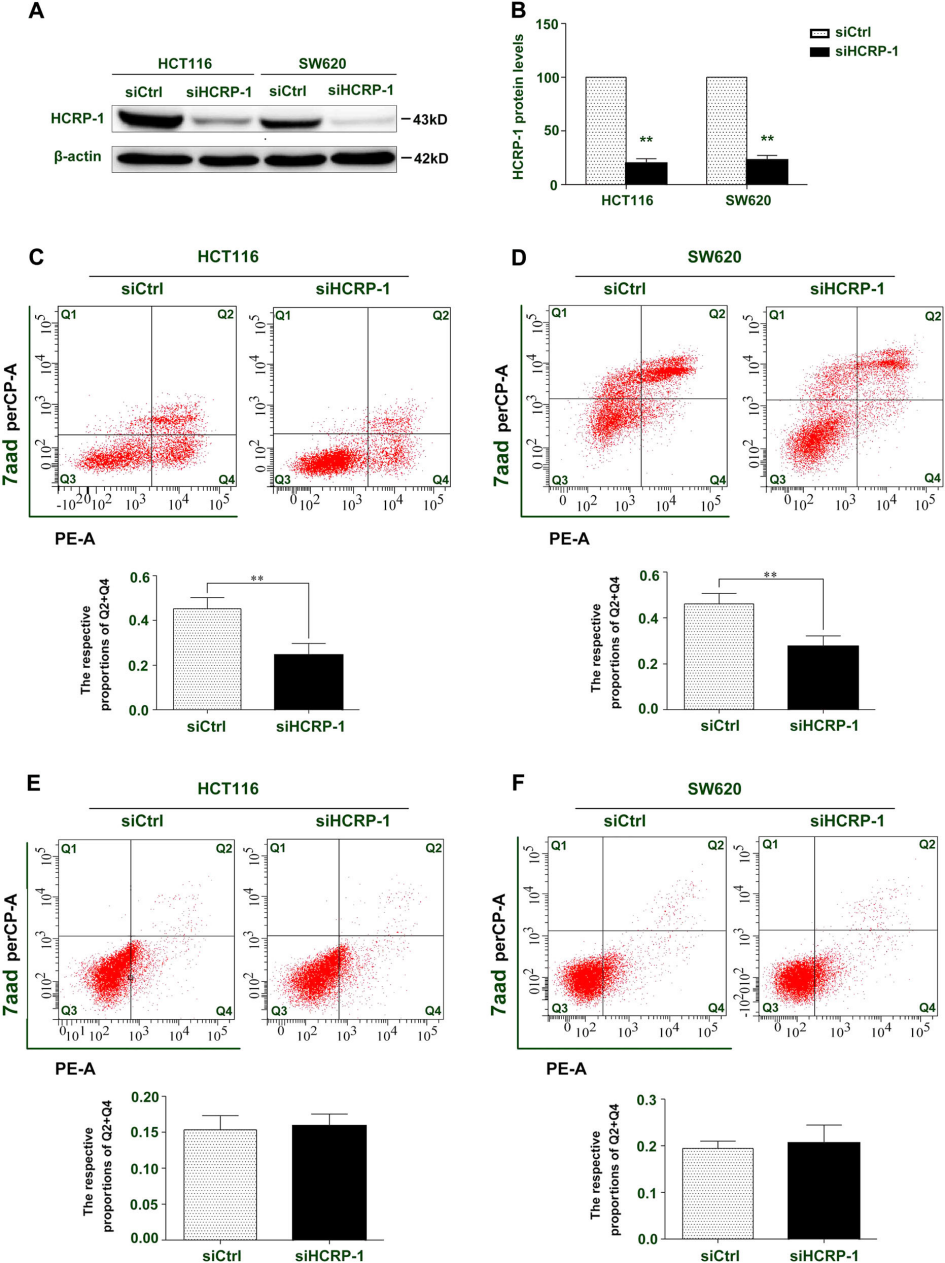
Inhibition of HCRP-1 attenuates colon cancer cell anoikis. **a**, **b** Cells were transfected with siRNA for HCRP-1 for 48 h, and then suspended in six-well plates with low-attachment surface. Cells that did not adhere were harvested and Western blot was employed to detect the relative protein expression of HCRP-1 in HCRP-1 siRNA group and control siRNA group. β-actin was used as a whole-cell protein internal control. Error bars indicate mean ± SD. **c**, **d** Transfected colon cancer cells were seeded in six-well plates with low-attachment surface. Cells that did not adhere were stained with Annexin V-PE/7-AAD at room temperature for 30 min, respectively. Then, cells were analyzed by FACScan flow cytometry. Viable cells (annexin V^−^/PI^−^), early apoptotic cells (annexin V^+^/PI^−^), late apoptotic cells (annexin V^−^/PI^+^), and necrotic cells (annexin V^+^/PI^+^) are located in the bottom left, bottom right, and top-right quadrants, respectively. The numbers in each quadrant represent the percentage of cells. The early apoptotic cells (annexin V^+^/PI^−^) and late apoptotic cells (annexin V^−^/PI^+^) were analyzed. Error bars indicate mean ± SD (*P* *<* 0.05). **e**, **f** Transfected colon cancer cells were harvested and stained with Annexin V-PE/7-AAD at room temperature for 30 min. Then, cells were analyzed by FACScan flow cytometry. Error bars indicate mean ± SD (*P *<* 0.05)

Anoikis plays an important role in preventing oncogenesis, particularly metastasis. To investigate whether HCRP-1 regulates anoikis in CRC, HCRP-1 protein was decreased with siRNAs (Fig. 2a, b). Anoikis assays showed that the rate of anoikis cells was significantly decreased following transfection with HCRP-1 siRNA (Fig. 2c, d). However, interference of HCRP-1 expression did not influence apoptosis in cancer cells (Fig. 2e, f). Therefore, our results showed that negative expression of HCRP-1 leads to anoikis resistance in CRC cells.

### Suppression of HCRP-1 attenuates BIM expression and activates the EGFR and AKT signaling pathway

**Fig. 3:**
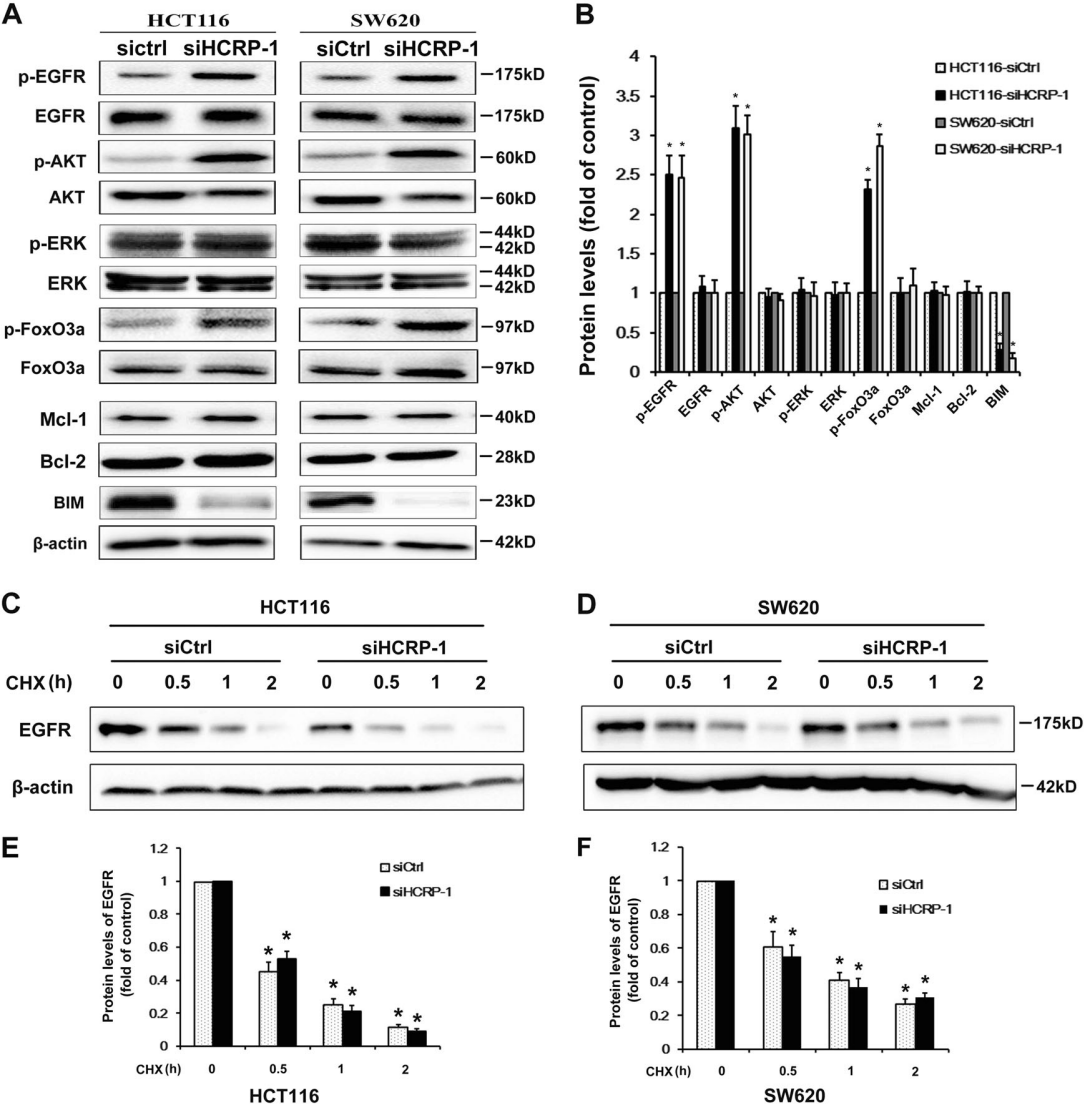
Inhibition of HCRP-1 activates the EGFR/AKT signaling pathway and suppresses BIM protein expression. **a**, **b** HCT116 and SW620 cells were transfected with si-HCRP-1 or control siRNA, and then cells were harvested and submitted to Western blot detection for the protein expression of BIM, FoxO3a, EGFR, AKT, ERK, Mcl-1, Bcl-2, and β-actin. Phosphorylated forms of EGFR, AKT, FoxO3a, and ERK were also detected by western blot with the corresponding antibodies. **c–f** Cells were treatedwith CHX for 0, 0.5 , 1, and 2 h after transfection with si-HCRP-1 or control siRNA for 48 h, lysates obtained from these cells were submitted to western blot detection for the protein expression of EGFR. Error bars indicate mean ± SD. Every experiment was repeated at least three times. *P < 0.05 vs. siCtrl group

As the intrinsic pathways of anoikis start from BIM, which is known as a proapoptotic factor, we first analyzed BIM expression by western blot and our data showed that HCRP-1 depletion reduced its protein expression. Besides BIM, we also detected the expression of other Bcl-2 family members, Mcl-1 and Bcl-2. The suppression of HCRP-1 did not change Mcl-1 and Bcl-2 protein levels (Fig. 3).

Constitutive activation of AKT and ERK1/2 signaling pathways could increase expression of BIM. Therefore, we took considerable interest to explore the levels of phosphorylated forms of AKT and ERK1/2. The results indicated that HCRP-1 downregulation significantly induced AKT phosphorylation, but did not affect p-ERK. Akt can regulate BIM expression transcriptionally through phosphorylation of FoxO3a. We examined the phosphorylation of FoxO3a and BIM mRNA levels. Data showed the elevated phosphorylation of FoxO3a and decreased BIM mRNA levels (Fig. 3a, b, SFig.1). Therefore, we concluded that anoikis progression regulated by HCRP-1 might be attributed to AKT/BIM signaling.

HCRP mediates the internalization and degradation of EGFR. We test the half-life of EGFR with CHX in both WT and HCRP knockdown cancer cells to clarify whether EGFR stability contributed to the activated EGFR–AKT pathway in HCRP knockdown cells. We observed that the stability was sustained in both cell lines (Fig. 3c–f). Ineffective receptor sorting could lead to phosphorylated EGFR receptor accumulation in cancer cells. Moreover, phosphorylated EGFR could trigger the activation of various pathways, such as the AKT signaling pathway. As HCRP-1 depletion activated the AKT pathway, we speculated that HCRP-1 might regulate EGFR activation, which acts upstream of the AKT pathway. We detected changes in EGFR activation and our results showed that EGFR phosphorylation (Tyr1173) was significantly elevated in HCRP-1-absent cells. On the other hand, HCRP-1 depletion induced EGFR phosphorylation in CRC cell lines, suggesting that HCRP-1 can negatively regulate EGFR activation (Fig. 3a, b).

### Silencing HCRP-1 promotes anoikis resistance through BIM

**Fig. 4:**
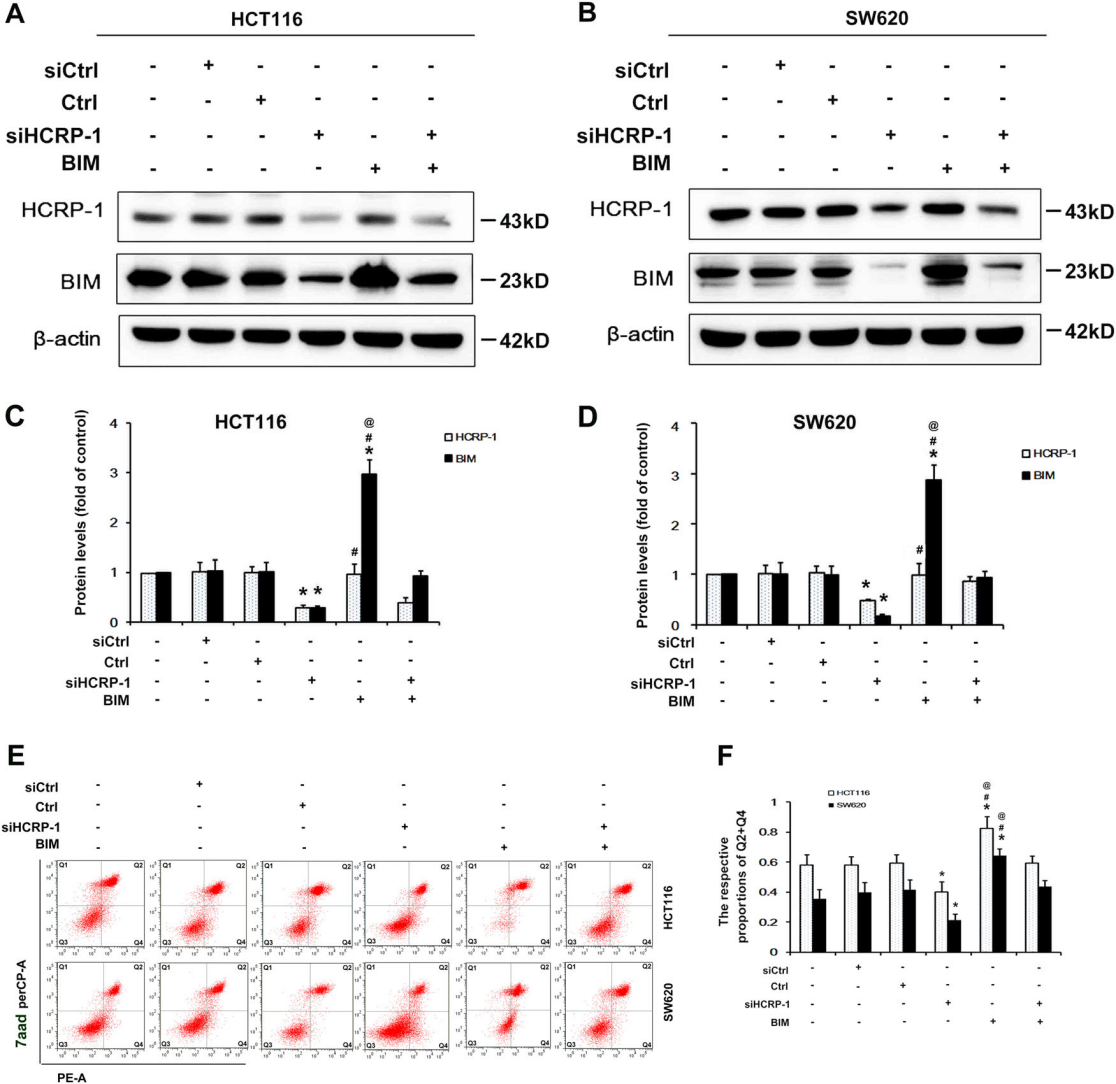
Silencing of HCRP-1 promotes anoikis resistance through BIM inhibition. **a–d** Cells were co-transfected with siRNA for HCRP-1 and/or BIM-expressing plasmids for 48 h, and then suspended in six-well plates with low-attachment surface. Cells that did not adhere were harvested and Western blot was employed to detect the expression of HCRP-1 and BIM. β-actin was used as a whole-cell protein internal control. Error bars indicate mean ± SD. **e**, **f** HCT116 and SW620 cells were co-transfected with HCRP-1 siRNA and/or BIM plasmids, and then suspended in six-well plates with low- attachment surface. Cells that did not adhere were analyzed by flow cytometry. Error bars indicate mean ± SD. *P *<* 0.05 vs. siCtrl or Ctrl group; ^#^*P* *<* 0.05 vs. siHCRP-1 group; ^@^*P* *<* 0.05 vs. siHCRP-1 + BIM group

To determine whether BIM is involved in anoikis resistance mediated by silencing HCRP-1, we transfected BIM plasmids in HCRP-1-silenced CRC cells, and then subjected them to Western blot and for anoikis detection. The data from Fig. 4a–d showed that BIM expression was increased after transfection with BIM plasmids in CRC cells, but could be inhibited by knockdown of HCRP-1 in HCT116 and SW620 cells. Subsequently, anoikis assay analysis revealed that overexpression of BIM could suppress anoikis resistance. The rate of anoikis was increased after forced BIM expression. However, it can be reversed by silencing HCRP-1. The anoikis cells were decreased after being given siRNA for HCRP-1 (Fig. 4e, f). These data suggested that overexpression of BIM could suppress the anoikis resistance mediated by silencing HCRP-1.

### Loss of HCRP-1 inhibits BIM protein levels via the AKT signaling pathway

**Fig. 5:**
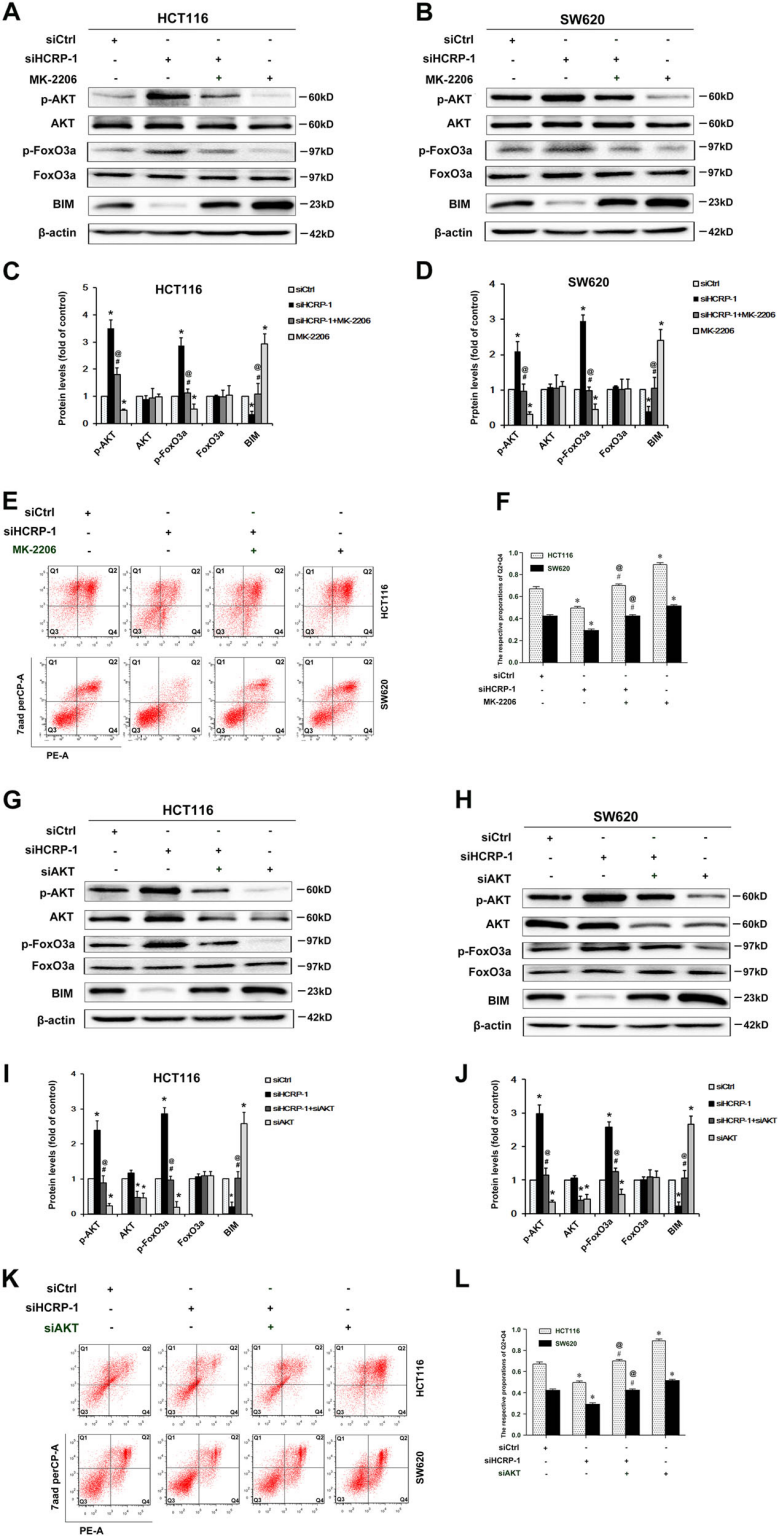
Inhibition of AKT inhibitor attenuates HCRP-1 depletion induced by AKT activation, upregulates BIM expression, and promotes anoikis. **a–d** HCT116 and SW620 cells were pretreated with HCRP-1 siRNA and the AKT inhibitor, MK-2206 (10 mM/L), and then suspended in six-well plates with low-attachment surface. The whole-cell lysates were analyzed for the protein levels of p-AKT, AKT, p-FoxO3a, FoxO3a, BIM, and β-actin by Western blot. **e**, **f** Cell anoikis was detected by flow cytometry in CRC cancer cells pretreated with HCRP-1 siRNA and/or MK-2206. **g–j** HCT116 and SW620 cells were co-transfected with HCRP-1 siRNA and/or AKT siRNA. Cells that did not adhere were harvested and western blot was employed to detect the expression of p-AKT, AKT, BIM, and β-actin. **k**, **l** Cell anoikis was detected by flow cytometry in colon cancer cells pretreated with HCRP-1 siRNA and/or AKT siRNA. Error bars indicate mean ± SD. Every experiment was repeated at least three times. *P < 0.05 vs. siCtrl group; ^#^*P* < 0.05 vs. siHCRP-1 group; ^@^*P* *<* 0.05 vs. MK2206 or siAKT group

To further confirm whether AKT phosphorylation induced by HCRP-1 suppression led to the negative expression of BIM in HCT116 and SW620 cells, we added the AKT-specific inhibitor, MK-2206 (10 mM/L) into CRC cells transfection with siRNA for HCRP-1. As expected, western blot results indicated that MK-2206 could significantly decrease AKT and FoxO3a activation and increase BIM expression in HCRP-1-knockdown cells (Fig. 5a–d). In addition, the rate of anoikis was obviously increased in HCRP-1-deficient cells treated with MK-2206 (Fig. 5e, f).

In addition, we determined the effect of HCRP-1 on BIM downregulation in HCRP-1-deficient cells transfected with siRNA for AKT. Similar results were found in HCRP-1-negative cells transfected with a specific siRNA for AKT. As shown in Fig. 5g–l, the expression of BIM and p-FoxO3a and the rate of anoikis were increased. These data provided sufficient evidence that depletion of HCRP-1 might downregulate BIM expression via activation of the AKT signaling pathway.

### Activation of the AKT signaling pathway depends on the accumulation of activated EGFR in HCRP-1-deficient CRC cells

**Fig. 6:**
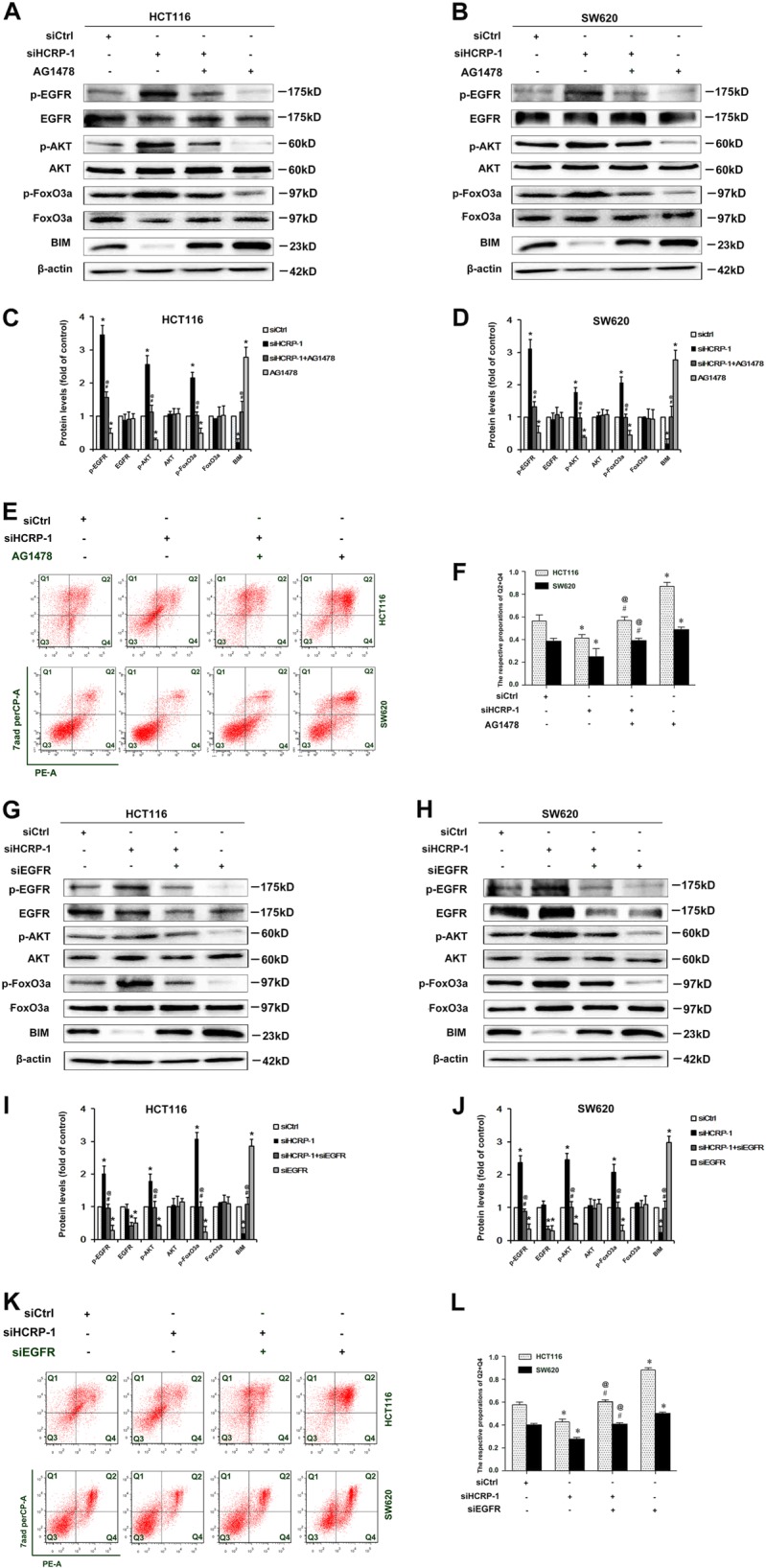
Suppression of EGFR blocks the loss of HCRP-1 induced by the AKT signaling pathway, upregulates BIM expression, and suppresses anoikis resistance. **a**, **b** HCT116 and SW620 cells were pretreated with HCRP-1 siRNA and the EGFR inhibitor, AG1478 (5 mM/L), and then suspended in six-well plates with low-attachment surface. The whole-cell lysates were analyzed for the protein levels of p-EGFR, EGFR, p-AKT, AKT, BIM, and β-actin by using western blot. **c**, **d** Cell anoikis was detected by flow cytometry. HCT116 and SW620 cells were pretreated with HCRP-1 siRNA and the EGFR inhibitor, AG1478 (5 mM/L), and then suspended in six-well plates with low- attachment surface. Cells that did not adhere were subjected to flow cytometry detection. **e** and f HCT116 and SW620 cells were co-transfected with HCRP-1 siRNA and/or EGFR siRNA. Levels of p-EGFR, EGFR, p-AKT, AKT, BIM, and β-actin in cells that did not adhere were determined by Western blot. **g**, **h** Cell anoikis was detected by flow cytometry in colon cancer cells pretreated with HCRP-1 siRNA and/or EGFR siRNA. Error bars indicate mean ± SD. Every experiment was repeated at least three times. *P < 0.05 vs. siCtrl group; ^#^*P* < 0.05 vs. siHCRP-1 group; ^@^*P* *<* 0.05 vs. AG1478 or siEGFR group

We then investigated whether the activation of AKT signaling induced by the negative expression of HCRP-1 was EGFR-dependent. The EGFR inhibitor, AG1478 (5 mM/L) was used to suppress EGFR activation. As shown in Fig. 6a–d, AG1478 obviously inhibited EGFR activation, the blocked AKT signaling pathway, and increased BIM expression in HCT116 and SW620 cells transfected with siRNA for HCRP-1. The rate of anoikis was also inhibited when cells were exposed to AG1478 (Fig. 6e, f). To further validate our hypothesis, siRNA for EGFR was used to attenuate EGFR expression. As shown in Fig. 6g–j, AKT signaling was blocked and the expression of BIM protein was increased in HCRP-1-knockdown cells transfected with siRNA for EGFR. Subsequently, anoikis assay analysis showed that the anoikis resistance was increased in CRC cells. The results were consistent with the treatment of AG1478 (Fig. 6k, l). These data collectively support our hypothesis that the activation of AKT signaling is dependent on accumulation of phosphorylated EGFR in HCRP-1-absent cells.

### Inhibition of HCRP-1 promotes tumor metastasis in vivo

**Fig. 7:**
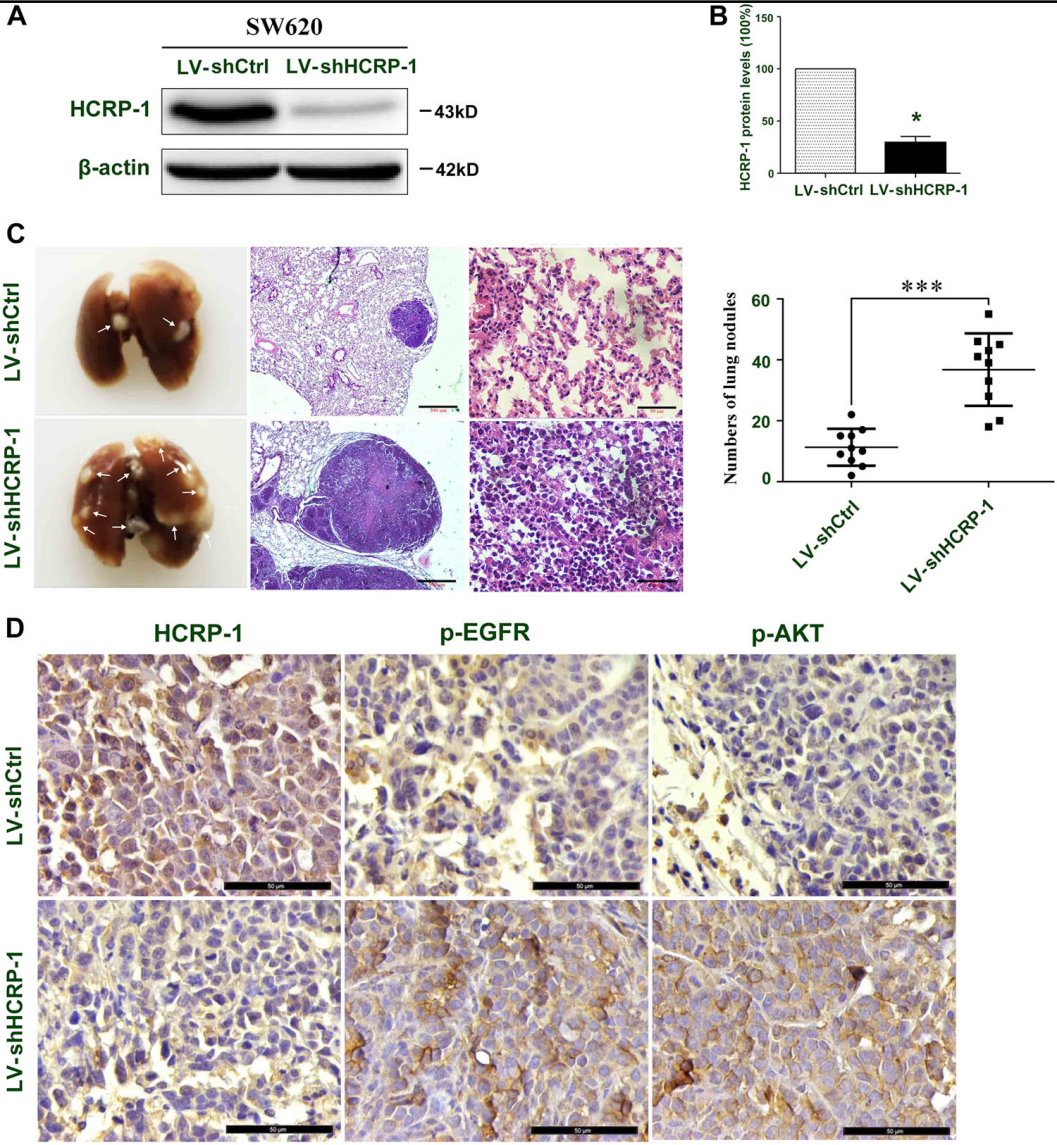
Loss of HCRP-1 induces tumor metastasis in vivo. **a**, **b** SW620 cells were stably transfected with shHCRP-1 lentivirus. The transfection efficiency was detected by Western blot; error bars indicate mean ± SD. ***P *<* 0.001 vs. LV-shCtrl group. **c** Number of metastatic nodules injected with SW620 cells stably transfected with HCRP-1 lentivirus compared with the control groups. Metastatic nodules were highlighted by white arrows; the corresponding hematoxylin/eosin-stained lung sections are shown. Error bars indicate mean ± SD. ***P *<* 0.001. **d** IHC detection of HCRP-1, p-EGFR, and p-AKT protein expression in xenograft tumors formed by SW620-shCtrl/shHCRP-1 cells in BALB/c female nude mice

Anoikis resistance is a critical factor for tumor metastasis. To determine the role of HCRP-1 in regulating anoikis in CRC cells, tail vein metastatic assay was used to analyze the effects of HCRP-1 depletion on the metastatic potency of SW620 cells in vivo. SW620 cells were infected with lentivirus, as described above and lentivirus fluorescence intensity was detected with an Olympus light microscope (Fig. 7a). We injected shHCRP-1-SW620 cells into the tail veins of the nude mice to establish a tumor metastasis model and compared numbers of lung nodules with those among control nude mice injected with shCtrl-SW620 cells. We found that the number of metastatic lung nodules in HCRP-1-knockdown mice was significantly higher than that of the control group (Fig. 7b, c).

In addition, the lung tissues were paraffin-embedded for subsequent analysis and the expression of HCRP-1, p-EGFR, and p-AKT was detected via immunohistochemistry staining (Fig. 7d). The results revealed that loss of HCRP-1 promoted tumor metastasis in vivo and activated the EGFR and AKT signaling pathway, which was consistent with the results obtained in in vitro experiments.

## Discussion

HCRP-1 was first cloned from hepatocellular carcinoma cell lines and was thought to be a growth inhibitory protein. The low expression of HCRP1 in hepatocellular carcinoma serves as an independent predictor for postoperative disease-free survival^13^. Since then, several studies have explored the role of HCRP-1 in tumorigenesis. Ectopic expression of HCRP-1 was observed in ovarian, breast, and oral and oropharyngeal cancer. Analysis of clinical tissue samples has provided evidence that the decrease in HCRP-1 protein expression is significantly associated with poor survival of these cancers^10–12^. More importantly, HCRP-1 affected cetuximab sensitivity and prognostic value of EGFR and HER2 in ovarian cancer^11^. Furthermore, HCRP1 could inhibit breast cancer metastasis by suppressing EGFR phosphorylation^12^. In our previous study, we found that the depletion of HCRP-1 obviously improved the migration and invasion abilities of renal cell carcinoma cells via EGFR–ERK signaling^14^. However, studies concerning HCRP-1 are still blank in CRC so far.

In the present work, we first found that HCRP1 protein was decreased or absent in CRC tissues. We also evaluated the association between HCRP-1 levels and clinicopathological features. Our findings provided evidence that low expression of HCRP-1 was associated with tumor size, pT status, TNM stage, and histological grade. Moreover, analysis of Cox proportional hazards regression indicated that HCRP-1 was an independent prognostic marker for CRC. Our clinical evidence supported the notion that decreased HCRP-1 expression can be considered as an important prognostic marker for CRC patients who are at high risk of unfavorable survival and may play a potential role in CRC metastasis.

Anoikis, a special programmed cell death, is due to the disengagement between cells and the extracellular matrix (ECM) or neighboring cells. The cells that lost contact with the neighboring cells or ECM will undergo apoptosis^17,18^. Once tumor cells develop a resistance to anoikis, they will extravasate to a distant location and metastasize. To determine whether HCRP-1 plays a role in cancer metastasis of CRC, we used siRNA to knock down endogenous HCRP-1 in HCT116 and SW620 cells, and found that decreased HCRP-1 expression was significantly correlated with anoikis resistance.

BIM, a member of the Bcl-2 protein family, is an essential initiator of anoikis^19^. Over the past decade, BIM has emerged as an essential proapoptotic protein for initiating the intrinsic anoikis pathway^18–21^. Downregulation of the BIM protein caused by some pathways might lead to anoikis resistance and tumor metastasis. Therefore, we analyzed BIM expression by western blot and found that HCRP-1 depletion reduced BIM protein expression. Moreover, other Bcl-2 protein family members are reported to participate in the regulation of anoikis. Mcl-1 degradation primes the cancer cells for Bax activation and anoikis^22^. Ubiquitin–proteasomal degradation of Bcl-2 sensitizes non-small-cell lung cancer cell anoikis^23^. To determine whether these proteins are involved in the anoikis resistance mediated by HCRP-1, we also examined the protein levels of Bcl-2 and Mcl-1. However, the inhibition of HCRP-1 did not change Bcl-2 and Mcl-1 protein expression. We further determined whether BIM is involved in anoikis resistance mediated by silencing HCRP-1. The results of western blot and anoikis assay displayed that forced BIM expression could suppress the anoikis resistance mediated by silencing HCRP-1. Herein, we concluded that silencing of HCRP-1 may lead to anoikis resistance by inhibiting the expression of anoikis-related protein BIM in CRC. However, the mechanisms of downregulation of BIM by HCRP-1 and its possible signal transduction pathway to regulate CRC cells anoikis are still not clear.

To fully elucidate the mechanisms involved in the depletion of HCRP-1-induced CRC cell anoikis resistance, we further investigated signaling pathways of BIM associated with anoikis. AKT is a serine/threonine protein kinase downstream target of the phosphatidylinositol 3-kinase (PI3K) signaling pathway, a central player in response to growth factors, which contributes to several cellular functions, including nutrient metabolism, cell growth, transcriptional regulation, and cell survival^24^. In a wide variety of cancers, AKT is frequently overactivated, contributing to malignancy and tumor aggressiveness^25^. The activated AKT pathway influences many factors involved in anoikis, either by transcription regulation or direct phosphorylation^26^. Luo et al. reported that enhanced ROS interacts with AKT/FoxO3a/BIM, which causes Akt inhibition and consequently nuclear accumulation of FoxO3a, thus facilitating transcription of the target gene BIM^27^. Except the AKT pathway, constitutive activation of ERK1/2 signaling pathways could lead to anoikis via increased expression of BIM^22^. Therefore, we evaluated the involvement of phosphorylated forms of AKT and ERK1/2. Our data showed that HCRP-1 downregulation significantly increased p-AKT and p-FoxO3a expression, but rarely had an impact on p-ERK in HCRP-1-depleted cells. Based on the above results, we assumed that the loss of HCRP-1 inhibits BIM protein levels by activating the AKT signaling pathway. To further confirm this assumption, the AKT-specific inhibitor MK-2206 and siRNA for AKT were used to abrogate the activation of AKT, and the results showed that BIM expression and anoikis were reversed by these pretreatments in HCT116 and SW620 cells. Therefore, depletion of HCRP-1 could downregulate BIM expression and result in anoikis resistance via the activation of the AKT signaling pathway.

Increasing evidence supports that the presence of aberrant signaling by EGFR is implicated in CRC progression^28^. The reason is that autophosphorylation of EGFR always leads to the activation of several pathways, including MAPK and PI3K–AKT. These pathways regulate cell proliferation, migration, metastasis, and evasion of apoptosis^29^. Phosphorylated EGFR receptor accumulation is caused by invalid receptor sorting in HCRP-1-depleted ovarian cancer cells^11^. Moreover, BIM protein expression is always downregulated in non-small-cell lung cancer carriers of EGFR mutations^30^. Therefore, we presumed that the activation AKT signaling induced by low expression of HCRP-1 depends on phosphorylated EGFR. In support of this, we used siRNA for HCPR-1 and found that HCRP-1 depletion obviously increased p-EGFR expression. Then the EGFR inhibitor AG1478 and siRNA for EGFR were used to attenuate EGFR expression in CRC cells. The results showed that p-AKT, p-FoxO3a and BIM expression, and anoikis resistance were reversed by these pretreatments in HCT116 and SW620 cells. As anoikis is important for disease and tumor metastasis^31^, we also carried out animal experiments. The results showed that reduced HCRP-1 expression promoted tumor metastasis and activated the EGFR and AKT signaling pathway, which were consistent with the results in vitro. These results support the hypothesis that the activation of AKT signaling depends on accumulation of phosphorylated EGFR in HCRP-1-knockdown CRC cells.

**Fig. 8:**
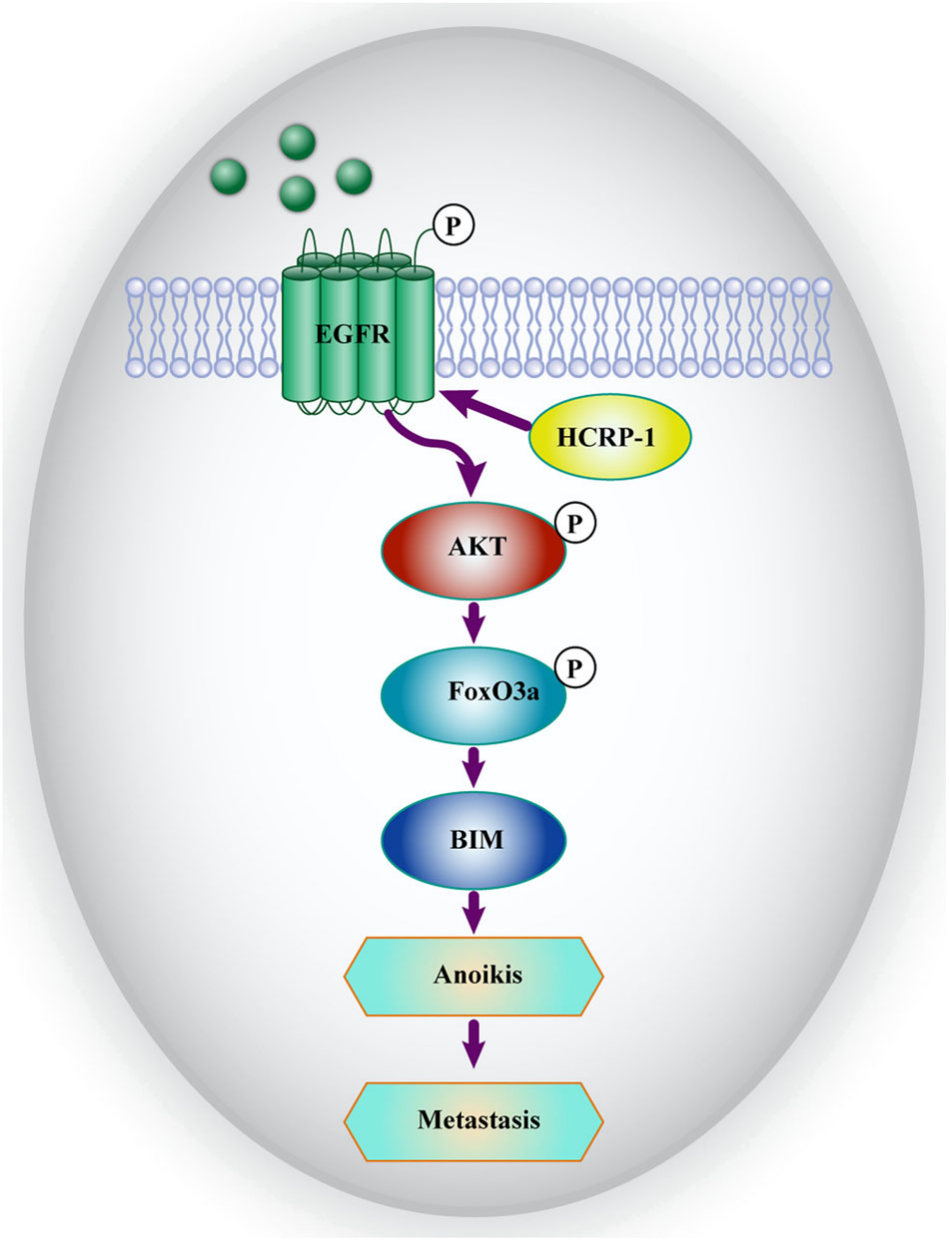
Hypothetic model of HCRP-1 depletion inhibits BIM expression via the EGFR–AKT signaling pathway

In general, this study provides evidence that HCRP-1 might be an independent prognostic factor for CRC. Moreover, HCPR-1 exerts an influence on cell anoikis through BIM regulation, which is the result of EGFR–AKT signaling pathway modulation. A hypothetic model is shown in Fig. 8. Thus, HCRP-1 might be considered as a promising novel therapeutic target for CRC.

## Electronic supplementary material

Supplementary figure legends

Supplementary materials
