# Supplementary material for: HCRP-1 regulates EGFR–AKT–BIM-mediated anoikis resistance and serves as a prognostic marker in human colon cancer
Source: Cell Death Dis. 2018 Dec 5;9(12):1176. doi: 10.1038/s41419-018-1217-2 (PMC6281589; doi:10.1038/s41419-018-1217-2)
Supplement: Supplementary file 1 — Supplementary figure legends [file 41419_2018_1217_MOESM1_ESM.docx]

**Supplementary Figure 1. Inhibition of HCRP-1 suppresses BIM mRNA expression. a and b** HCT116 and SW620 cells were transfected with si-HCRP-1 or control siRNA, then cells were harvested and submitted to PCR detection for the mRNA expression of HCRP-1 and BIM. **P* < 0.05 versus siCtrl group.
