# Supplementary material for: HCRP-1 regulates EGFR–AKT–BIM-mediated anoikis resistance and serves as a prognostic marker in human colon cancer
Source: Cell Death Dis. 2018 Dec 5;9(12):1176. doi: 10.1038/s41419-018-1217-2 (PMC6281589; doi:10.1038/s41419-018-1217-2)
Supplement: Supplementary file 2 — Supplementary materials [file 41419_2018_1217_MOESM2_ESM.docx]

Material and methods

**RNA extraction and real-time PCR**

RNA extraction was carried out using Trizol (Invitrogen) according to the manufacturer’s instructions. Real-time PCR amplification of HCRP-1, BIM and GAPDH was performed for 30 s at 95°C, followed by 40 cycles at 95°C for 5 s and annealing at 60°C for 34 s using an ABI PRISM 7500 Sequence Detection System (New York, USA). Target mRNA levels were calculated based on the CT method, normalized to GAPDH, and are expressed as a ratio of the percentage of gene copies to the GAPDH control. All reactions were run in triplicate. The primer sequences for each gene are as follows:

HCRP-1 Forward, 5’-CGTGGACCCGAGGAGGATGAGCTGG-3’,

Reverse, 5’-CTATAGTGGAGCATGAAATTGGCTG3’;

BIM Forward, 5’-CTATAGTGGAGCATGAAATTGGCTG 3’,

Reverse, 5’- CTATAGTGGAGCATGAAATTGGCTG-3’;

GAPDH Forward, 5’-TGAAGGTCGGAGTCAACGGATT-3’,

Reverse, 5’-CCTGGAAGATGGTGATGGGATT-3’.
